# Supplementary material for: CO electrolysis to multicarbon products over grain boundary-rich Cu nanoparticles in membrane electrode assembly electrolyzers
Source: Nat Commun. 2024 May 30;15:4603. doi: 10.1038/s41467-024-49095-2 (PMC11139892; doi:10.1038/s41467-024-49095-2)
Supplement: Supplementary file 1 — Supplementary Information [file 41467_2024_49095_MOESM1_ESM.pdf]

Supplementary Information for

CO electrolysis to multicarbon products over grain boundary-rich Cu nanoparticles in membrane electrode assembly electrolyzers

Hefei Li[1,2,#], Pengfei Wei[1,#], Tianfu Liu[1,#], Mingrun Li[1], Chao Wang[1], Rongtan Li[1,2], Jinyu Ye[3], Zhi-You Zhou[3], Shi-Gang Sun[3], Qiang Fu[1], Dunfeng Gao[1]*, Guoxiong Wang[1]*, Xinhe Bao[1]

[1]State Key Laboratory of Catalysis, Dalian National Laboratory for Clean Energy, *i*ChEM (Collaborative Innovation Center of Chemistry for Energy Materials), Dalian Institute of Chemical Physics, Chinese Academy of Sciences, Dalian 116023, China.

[2]University of Chinese Academy of Sciences, Beijing 100049, China.

[3]State Key Laboratory of Physical Chemistry of Solid Surfaces, *i*ChEM, College of Chemistry and Chemical Engineering, Xiamen University, Xiamen 361005, China.

[#]These authors contributed equally: Hefei Li, Pengfei Wei, Tianfu Liu.

*Corresponding authors. Emails: dfgao@dicp.ac.cn; wanggx@dicp.ac.cn

Table of Contents

Supplementary Notes 1−2 .... S2

Supplementary Figures 1−39 .... S8

Supplementary Tables 1−9 .... S43

Supplementary Notes

The techno-economic assessment (TEA) and CO_2 emission of the CO electrolysis process were calculated according to previous method[62–67]. Here we took the calculation at 4.5 A cm^{-2} for example, and the TEA and CO_2 emission based on the performance data at 1.0 and 3.0 A cm^{-2} were also calculated with the same method. These results were summarized in Supplementary Figure 39.

Supplementary Note 1. Techno-economic assessment (TEA) for CO electrolysis

The TEA of the CO electrolysis press was calculated based on the performance data acquired at an applied current density of 4.5 A cm^{-2} in the 4-cm^2 electrolyzer using previously reported parameters[62–65].

Input parameters

For costs of CO electrolysis, the following input parameters were used (Supplementary Table 1). Here taking the data at 4.5 A cm^{-2} for example, the calculation was operated under the conditions that CO electrolysis at 4.5 A cm^{-2} and a cell voltage of 2.74 V with a conversion rate of 84.2%.

Supplementary Table 1 | Standard values at 25 °C and market price of C_{2+} products from previous reports.

Compound	FE (%)	Selectivity (%)	n (e^-)	M (g mol^{-1})	Density (kg m^{-3})	Market price (USD kg^{-1})
**C_2H_4**	44.3	42.34	8	28.06	-	1.30 [62]
**n-C_3H_7OH**	5.4	5.18	12	60.1	804	1.435 [62]
**C_2H_5OH**	25.7	24.56	8	46.07	789.3	1.003 [62]
**CH_3COOH**	14.5	27.89	4	60.05	1050	1.2 [64]
**H_2**	10.1	-	-	2.01	-	1 [65]
**CO**	-	-	-	28.01	-	0.44 [64]
**H_2O**	-	-	-	18.01	997	0.00143 [62]

First, assuming a C_2H_4 production rate of 100,000 $kg\ day^{-1}$, the corresponding C_2H_4 partial current is:

$$100000\frac{kg}{day} * \frac{day}{86400s} * \frac{1000g}{kg} * \frac{mol}{28.06\ g} * 8\ e^{-} * 96485\frac{C}{mol} = 31838190.65\ \text{A}$$

The required total current is:

$$31838190.65\ \text{A} * \frac{1}{0.443} = 71869504.8\ \text{A}$$

The electrolyzer area needed is the total current divided by the current density:

$$71869504.8\ \text{A} * \frac{1}{4.5\ A/cm^2} * \frac{m^2}{10^4\ cm^2} = 1597.1\ m^2$$

The required daily power is defined as:

$$2.74\ \text{V} * 71869504.8\ \text{A} * \frac{W}{10^6 MW} = 196.9\ \text{MW}$$

The total required daily CO flow rate with a 42.34% selectivity of C_2H_4 is defined as:

$$31838190.65\ \text{A} * \frac{2}{8e^-} * \frac{1}{96485\ C/mol} * \frac{0.028\ kg}{mol} * \frac{86400\ s}{day} * \frac{1}{0.4234} = 471356.5\ kg\ day^{-1}$$

The inlet CO flow rate with 84.2% single-pass conversion is defined as:

$$471356.5\ \frac{kg}{day} * \frac{day}{24\ h} * \frac{1}{0.842} = 23325.2\ kg\ h^{-1}$$

The outlet CO flow rate is defined as:

$$23325.2\ \frac{kg}{h} * (1\text{-}0.842) * \frac{1000\ g}{kg} * \frac{mol}{28.01\ g} * \frac{24.46\ L}{mol} * \frac{m^3}{1000L} = 3218.3\ m^3\ h^{-1}$$

The outlet H_2 flow rate is:

$$71869504.8\ \text{A} * 0.101 * \frac{1}{2e^-} * \frac{1}{96485\ C/mol} * \frac{24.46\ L}{mol} * \frac{m^3}{1000L} * \frac{3600\ s}{h} = 3312.3\ m^3\ h^{-1}$$

$$71869504.8\ \text{A} * 0.101 * \frac{1}{2e^-} * \frac{1}{96485\ C/mol} * \frac{3600\ s}{h} * \frac{24\ h}{day} * \frac{2.01\ g}{mol} * \frac{1\ kg}{1000\ g} = 6532.6\ kg\ day^{-1}$$

The outlet C_2H_4 flow rate is:

$$71869504.8\ \text{A} * 0.443 * \frac{1}{8e^-} * \frac{1}{96485\ C/mol} * \frac{24.46\ L}{mol} * \frac{m^3}{1000L} * \frac{3600\ s}{h} = 3632.1\ m^3\ h^{-1}$$

The total outlet gas flow rate (Cathode) is:

$$3218.3\ \frac{m^3}{h} + 3312.3\ \frac{m^3}{h} + 3632.1\ \frac{m^3}{h} = 10162.7\ m^3\ h^{-1}$$

The outlet C_2H_5OH flow rate is:

$$71869504.8 \text{ A} * 0.257 * \frac{1}{8e^-} * \frac{1}{96485\ C/mol} * \frac{46.07\ g}{mol} * \frac{m^3}{0.7893g} * \frac{1000\ L}{m^3} * \frac{60\ s}{min} = 83.8\ L\ min^{-1}$$

$$71869504.8 \text{ A} * 0.257 * \frac{1}{8e^-} * \frac{1}{96485\ C/mol} * \frac{46.07\ g}{mol} * \frac{kg}{1000g} * \frac{86400\ s}{day} = 95248.9\ kg\ day^{-1}$$

The outlet n-C_3H_7OH flow rate is:

$$71869504.8 \text{ A} * 0.054 * \frac{1}{12e^-} * \frac{1}{96485\ C/mol} * \frac{60.1\ g}{mol} * \frac{m^3}{0.804g} * \frac{1000\ L}{m^3} * \frac{60\ s}{min} = 12.25\ L\ min^{-1}$$

$$71869504.8 \text{ A} * 0.054 * \frac{1}{12e^-} * \frac{1}{96485\ C/mol} * \frac{60.1\ g}{mol} * \frac{kg}{1000g} * \frac{86400\ s}{day} = 14182.2\ kg\ day^{-1}$$

The outlet CH_3COOH flow rate is:

$$71869504.8 \text{ A} * 0.145 * \frac{1}{4e^-} * \frac{1}{96485\ C/mol} * \frac{60.05\ g}{mol} * \frac{m^3}{1.05g} * \frac{1000\ L}{m^3} * \frac{60\ s}{min} = 92.65\ L\ min^{-1}$$

$$71869504.8 \text{ A} * 0.145 * \frac{1}{4e^-} * \frac{1}{96485\ C/mol} * \frac{60.05\ g}{mol} * \frac{kg}{1000g} * \frac{86400\ s}{day} = 140094\ kg\ day^{-1}$$

It is assumed the anolyte is recycled until a steady-state volume concentration of 10% is reached. Then the continuous outlet anolyte flow rate is:

$$(83.8\ \frac{L}{min} + 12.25\ \frac{L}{min} + 92.65\ \frac{L}{min}) \div 0.1 = 1887\ L\ min^{-1}$$

The water flow rate for the anodic OER reaction is:

$$71869504.8 \text{ A} * \frac{1}{4e^-} * \frac{1}{96485\ C/mol} * \frac{0.018\ kg}{mol} * \frac{m^3}{997kg} * \frac{1000\ L}{m^3} * \frac{86400\ s}{day} = 290479.8\ L\ day^{-1}$$

Capital cost

1. Electrolyzer Cost

$$1597.1\ m^2 * 919.7\ \frac{USD}{m^2} = 1468853 \text{ USD}$$

2. Balance of Plant

$$1468853 \text{ USD} * \frac{0.35}{0.65} = 790920.8 \text{ USD}$$

3. PSA (Pressure swing adsorption)

$$1989043 \text{ USD} * \left(\frac{10162.7\,\frac{m^3}{h}}{1000\,\frac{m^3}{h}}\right)^{0.7} = 10083638.2 \text{ USD}$$

4. Distillation capital cost

$$4162240 \text{ USD} * \left(\frac{838\frac{L}{min}+122.4\,\frac{L}{min}}{1000\,\frac{L}{min}}\right)^{0.7} + 6896190 \text{ USD} * \left(\frac{926.5\,\frac{L}{min}}{1000\,\frac{L}{min}}\right)^{0.7} = 10584091.46 \text{ USD}$$

So the sum of capital costs is 22927503.5 USD.

Operation cost

1. Electricity

$$196.9 \text{ MW} * \frac{1000\,kW}{MW} * \frac{24\,h}{day} * \frac{0.02\,USD}{kWh} = 90970\ \ USD\ day^{-1}$$

2. Maintenance for 350 days/year operation

$$1468853 \text{ USD} * 0.025 * \frac{1\,year}{350\,days} = 104.9\ \ USD\ day^{-1}$$

3. PSA

$$10162.7\ \frac{m^3}{h} * 0.25\ \frac{kWh}{m^3} * 0.02\ \frac{USD}{kWh} * \frac{24\,h}{day} = 1219.5\ \ USD\ day^{-1}$$

4. Distillation operating cost

$$\frac{9895\text{ USD}}{day} * \left(\frac{838\,\frac{L}{min}+122.4\frac{L}{min}}{1000\,\frac{L}{min}}\right)^{0.7} + \frac{32037\text{ USD}}{day} * \left(\frac{926.5\,\frac{L}{min}}{1000\,\frac{L}{min}}\right)^{0.7} = 39991.5\ \ USD\ day^{-1}$$

5. CO Purchase

$$471356.5\ \frac{kg}{day} * \frac{0.44\text{ USD}}{kg} = 207396\ \ USD\ day^{-1}$$

6. Water

$$71869504.8 \text{ A} * \frac{1}{4e^-} * \frac{1}{96485\ C/mol} * \frac{0.018\,kg}{mol} * \frac{86400\,s}{day} * \frac{0.00143\text{ USD}}{kg} = 414.4\ \ USD\ day^{-1}$$

7. Cell Compartment Replacement (Every 7 year)

$$1468853 \text{ USD} * 0.15 * \frac{1\,year}{365\,days} * \frac{1}{7\,years} = 86.2\ \ USD\ day^{-1}$$

8. MEA Replacement

$$1180\ \frac{USD}{m^2} * 1597.1\ \ m^2 * \frac{1\,year}{365\,days} = 5163.2\ \ USD\ day^{-1}$$

So the sum of operating costs is:

$$(90970\ +\ 104.9\ +\ 1219.5\ +\ 39991.5\ +\ 207396\ + 414.4 + 86.2\ + 5163.2\,)\ \frac{\text{USD}}{day} =$$

$$348899.5\ \ USD\ day^{-1}$$

The market price of pure C_2H_4, C_2H_5OH, C_3H_7OH, CH_3COOH, H_2 is reported as $\frac{1.30\text{ USD}}{kg}$, $\frac{1.003\text{ USD}}{kg}$, $\frac{1.435\text{ USD}}{kg}$, $\frac{1.2\text{ USD}}{kg}$, $\frac{1\text{ USD}}{kg}$.

So the product income is:

$$\frac{100000\text{ kg}}{day} * \frac{1.3\text{USD}}{kg} + \frac{95248.9\text{ kg}}{day} * \frac{1.003\text{ USD}}{kg} + \frac{14182.2\text{ kg}}{day} * \frac{1.435\text{ USD}}{kg} + \frac{140094\text{ kg}}{day} * \frac{1.2\text{ USD}}{kg}$$

$$+ \frac{6532.6\text{ kg}}{day} * \frac{1\text{USD}}{kg} = 420531.5\ \ USD\ day^{-1}$$

The yearly profit is given by the product income minus the sum of operating costs:

$$(420531.5\ \frac{USD}{day} - 348899.5\ \frac{USD}{day}) * \frac{350\,day}{year} = 25071209.49\ \ USD\ year^{-1}$$

Finally, the payback time should be:

$$22927503.5 \text{ USD (capital cost)} \div 25071209.49\ \frac{USD}{year} = 0.91\ \ year$$

Supplementary Note 2. Carbon footprint analysis

Assuming a C_2H_4 production rate of 1000 $kg\ day^{-1}$, the required daily electricity is:

$$1.969 \text{ MW} * \frac{1000\,kW}{MW} * \frac{24\,h}{day} = 47261.3 \text{ kWh}$$

So the energy consumption of C_2H_4 is 47261.3 $KWh/t_{C_2H_4}$ in CO electrolysis process.

The energy consumption of PSA process is:

$$101.6\ \frac{m^3}{h} * 0.25\ \frac{kWh}{m^3} * \frac{24\,h}{day} = 609.7\ \ KWh/t_{C_2H_4}$$

So the total energy consumption of CO electrolysis and PSA process is $47871.1 KWh/t_{C_2H_4}$.

Assuming that wind power is used in these processes, the carbon dioxide emission is 0.02 kg/KWh[62].

Thus, the total carbon dioxide emission during the two process is:

$$0.02\ \frac{kg}{kWh}\ *\ 47871.1\ \text{kWh}\ *\ \frac{1\,t}{1000\,kg} = 0.95\ \ t/t_{C_2H_4}$$

As reported[66,67], the total carbon dioxide emission during Fischer–Tropsch synthesis and separation process is 5.3 $t/t_{C_2H_4}$.

Hence, CO_2 emission is reduced by 82% with a value of 4.35 $t/t_{C_2H_4}$ in CO electrolysis process.

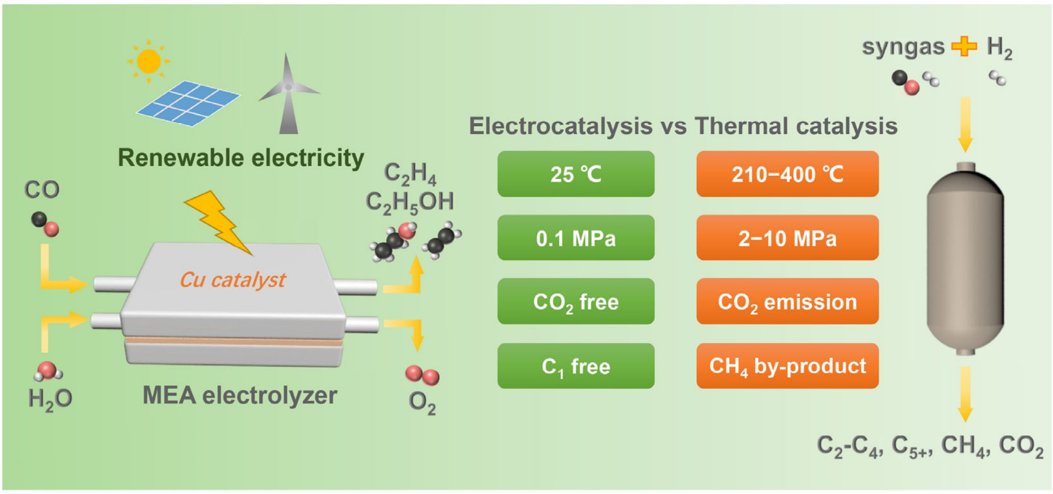

Supplementary Figure 1 | CO conversion to valuable chemicals via electrocatalysis and thermal catalysis.

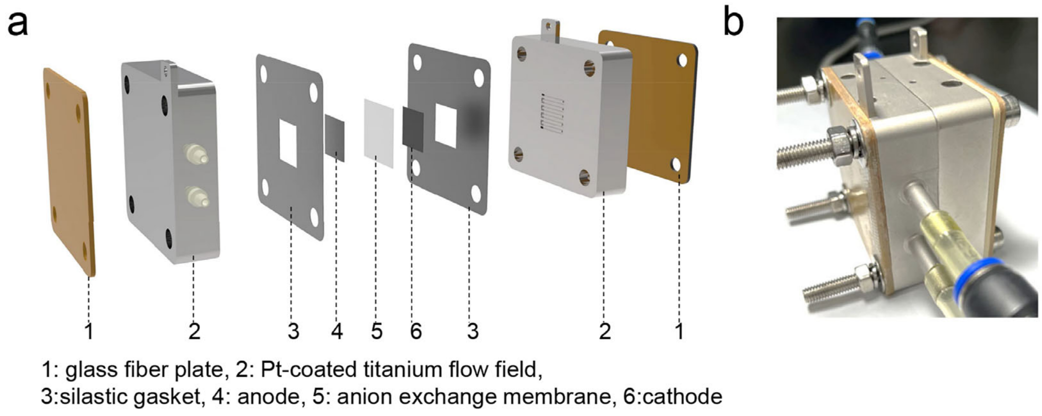

Supplementary Figure 2 | Schematic (a) and photograph (b) of home-made MEA electrolyzer with an electrode area of 4 cm^2 for CO electrolysis.

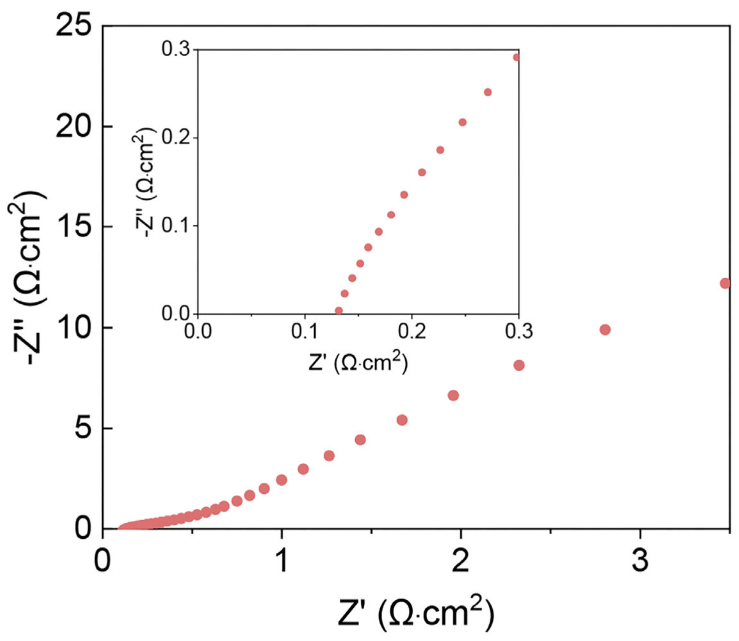

Supplementary Figure 3 | Electrochemical impedance spectroscopy (EIS) of MEA electrolyzer with Cu-nc catalyst measured in 0.5 M KOH at open circuit potential after electrolysis at 5.0 A cm^{-2} for 10 min. Source data are provided as a Source Data file.

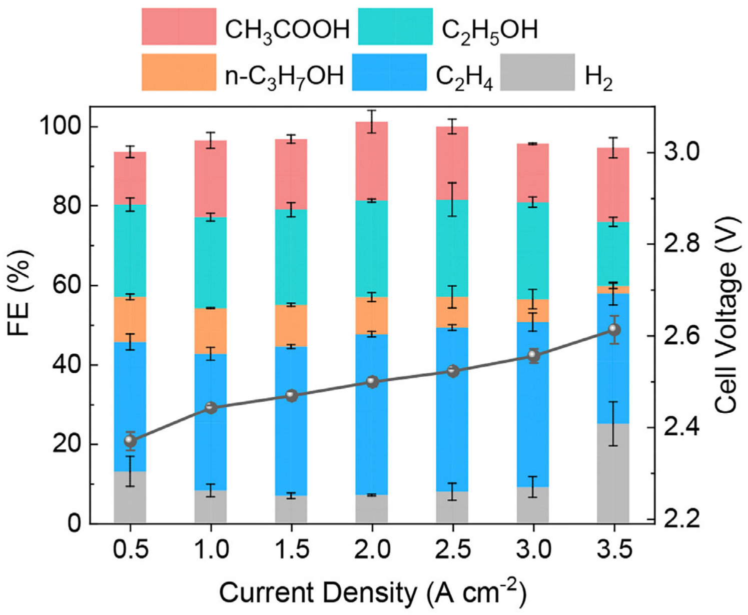

Supplementary Figure 4 | CO electrolysis performance of commercially available Cu nanoparticle catalyst measured in 0.5 M KOH (5.0 mL min^{-1}) under CO feed (80 mL min^{-1}), with a mass loading of 2.0 mg cm^{-2} in the electrode. Source data are provided as a Source Data file.

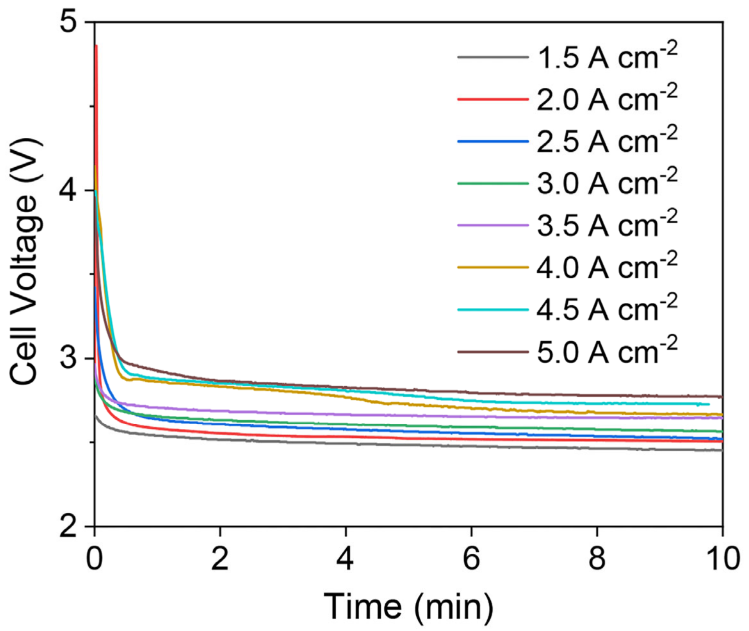

Supplementary Figure 5 | Chronopotentiometric profiles at high current densities. Source data are provided as a Source Data file.

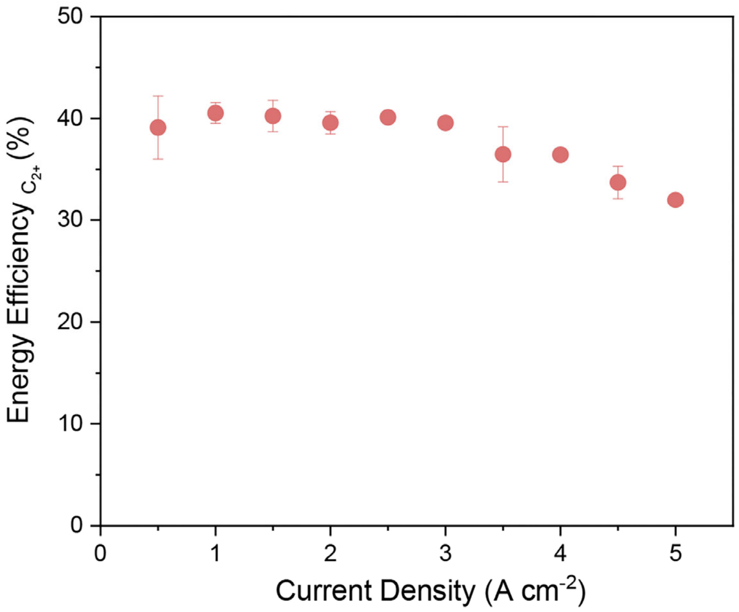

Supplementary Figure 6 | Energy efficiencies of C_{2+} products as a function of current density over Cu-nc catalyst. The error bars represent standard error of the mean and are made based on three fully separate and identical measurements. Source data are provided as a Source Data file.

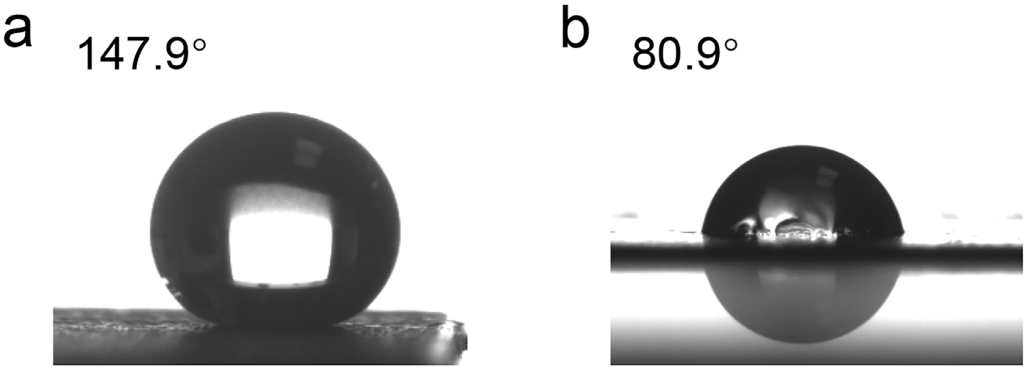

Supplementary Figure 7 | Contact angle measurement on Cu-nc electrode in the as-prepared state (a) and after stability test at 1.0 A cm^{-2} for 150 h (b).

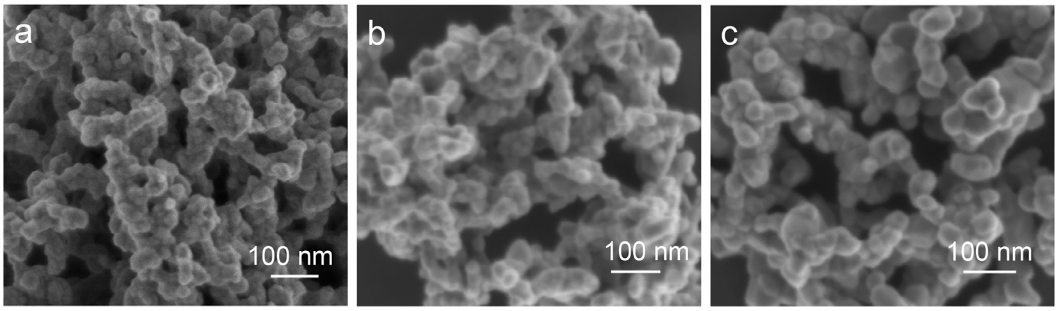

Supplementary Figure 8 | SEM images of (a) Cu-350, (b) Cu-450, and (c) Cu-550 catalysts in their as-prepared state.

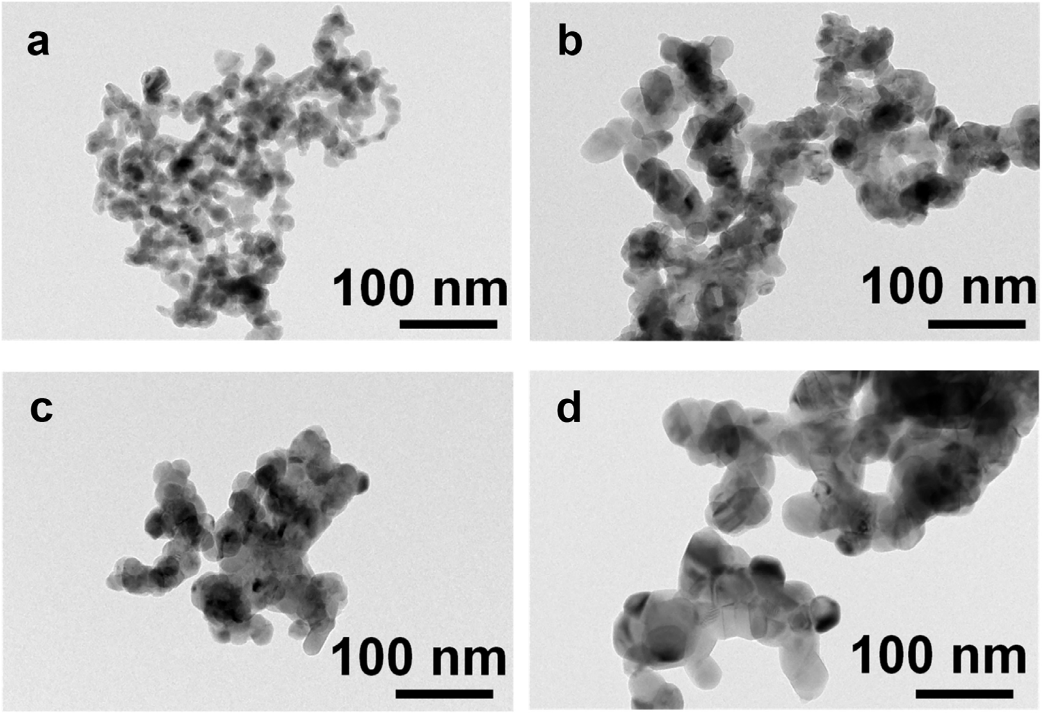

Supplementary Figure 9 | TEM images of (a) Cu-nc, (b) Cu-350, (c) Cu-450, and (d) Cu-550 catalysts in their as-prepared states.

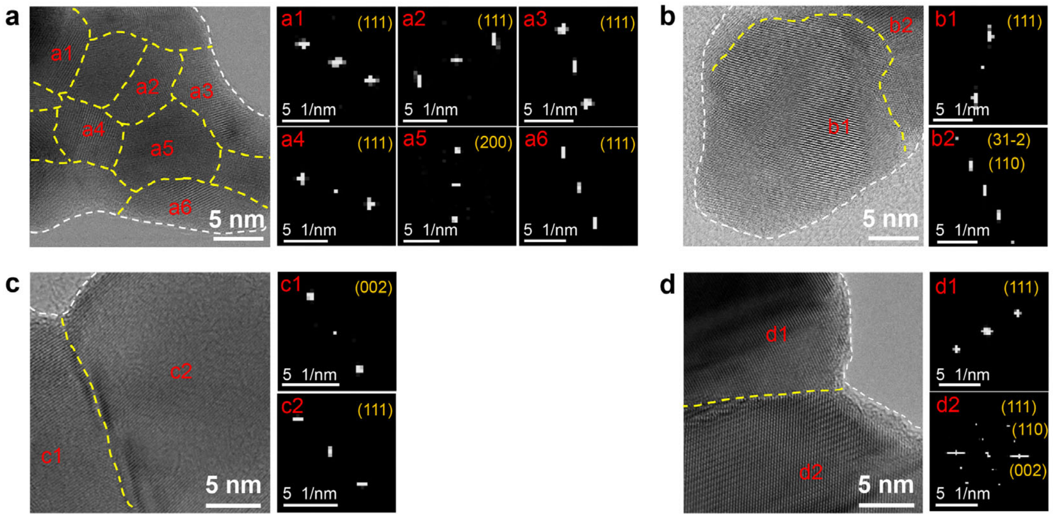

Supplementary Figure 10 Representative HRTEM images and the corresponding of FFT patterns acquired from each grain over as-prepared (a) Cu-nc, (b) Cu-350, (c) Cu-450, and (d) Cu-550 catalysts.

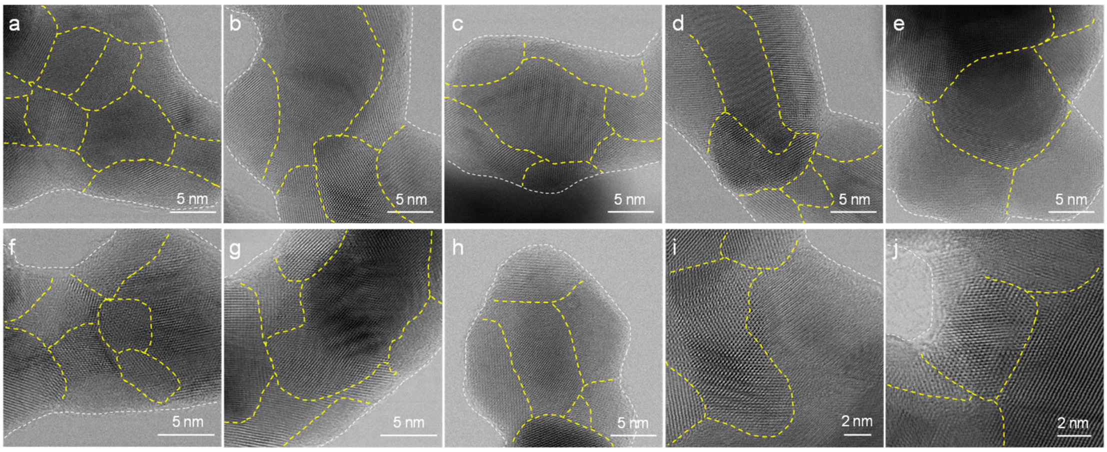

Supplementary Figure 11 | (a–j) TEM images of Cu-nc catalyst in their as-prepared state. GBs are considered as the border of two regions with different lattice orientations and are marked with yellow dashed lines in the TEM images.

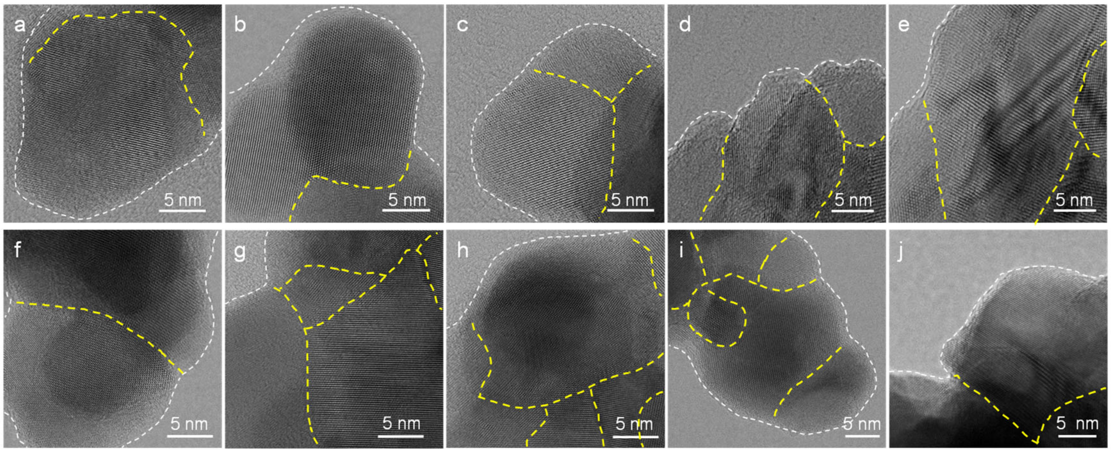

Supplementary Figure 12 | (a–j) TEM images of Cu-350 catalyst in their as-prepared state. GBs are considered as the border of two regions with different lattice orientations and are marked with yellow dashed lines in the TEM images.

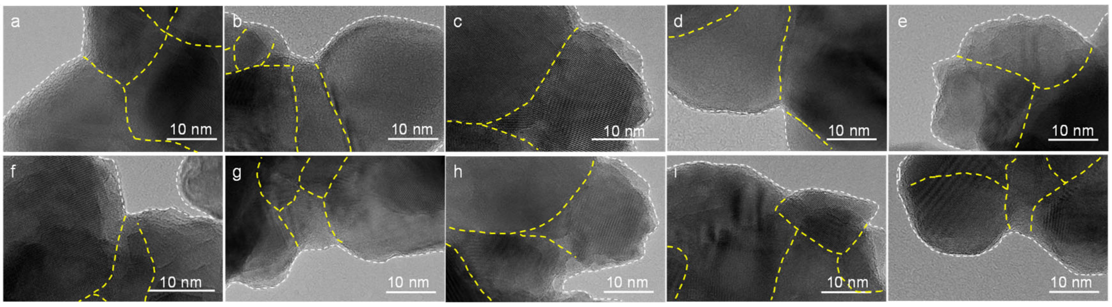

Supplementary Figure 13 | (a–j) TEM images of Cu-450 catalyst in their as-prepared state. GBs are considered as the border of two regions with different lattice orientations and are marked with yellow dashed lines in the TEM images.

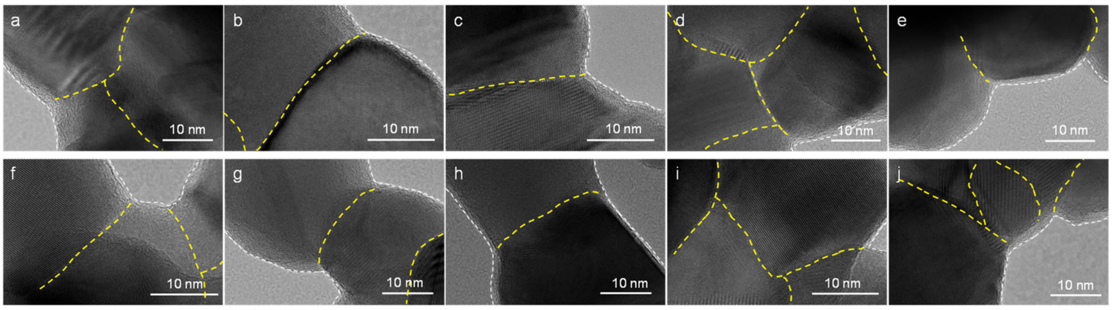

Supplementary Figure 14 | (a–j) TEM images of Cu-550 catalyst in their as-prepared state. GBs are considered as the border of two regions with different lattice orientations and are marked with yellow dashed lines in the TEM images.

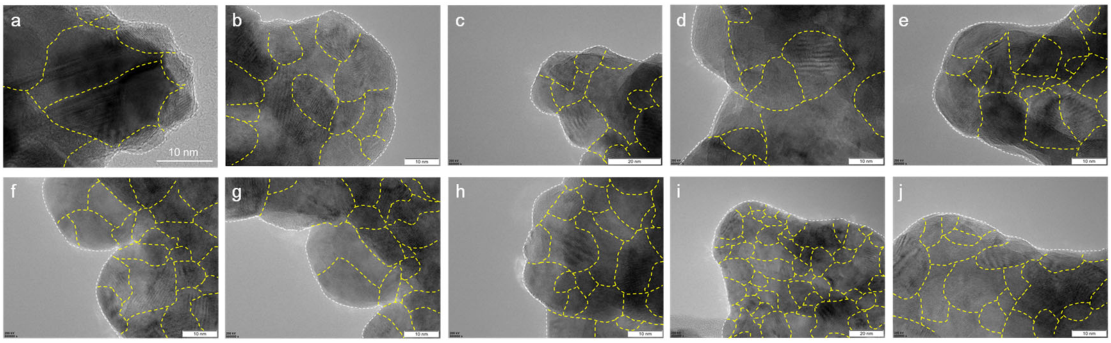

Supplementary Figure 15 | (a–j) TEM images of Cu-nc catalyst after CO electrolysis. GBs are considered as the border of two regions with different lattice orientations and are marked with yellow dashed lines in the TEM images.

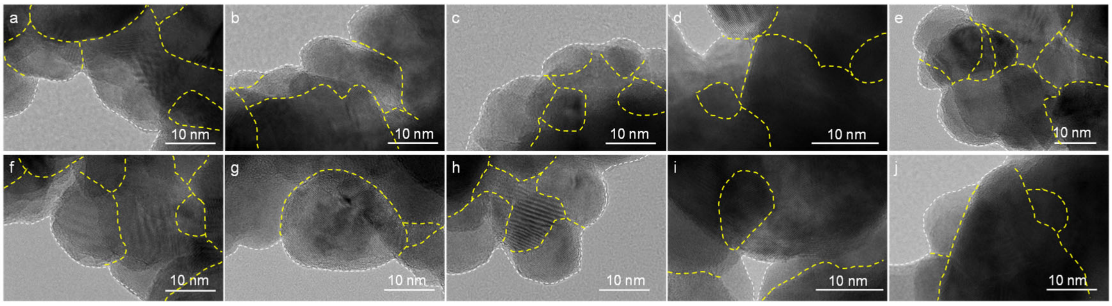

Supplementary Figure 16 | (a–j) TEM images of Cu-350 catalyst after CO electrolysis. GBs are considered as the border of two regions with different lattice orientations and are marked with yellow dashed lines in the TEM images.

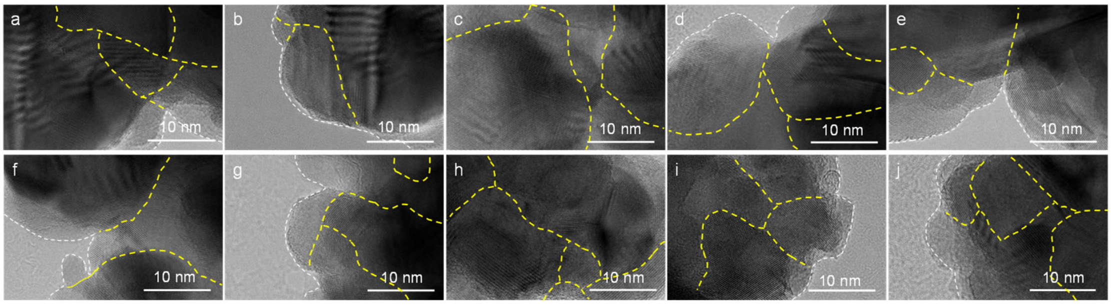

Supplementary Figure 17 | (a–j) TEM images of Cu-450 catalyst after CO electrolysis. GBs are considered as the border of two regions with different lattice orientations and are marked with yellow dashed lines in the TEM images.

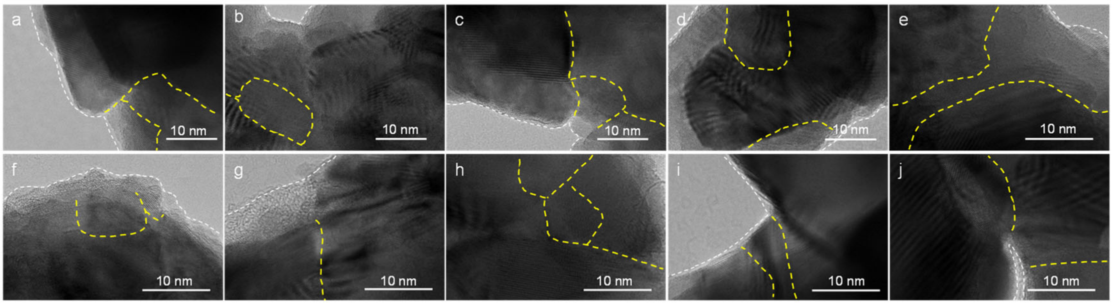

Supplementary Figure 18 | (a–j) TEM images of Cu-550 catalyst after CO electrolysis. GBs are considered as the border of two regions with different lattice orientations and are marked with yellow dashed lines in the TEM images.

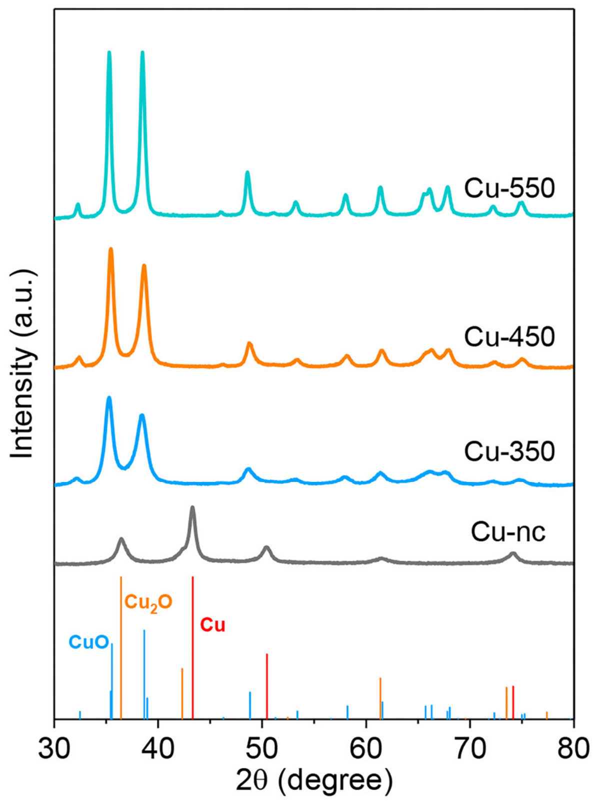

Supplementary Figure 19 | XRD patterns of as-prepared Cu-nc and Cu-*x* catalysts. Source data are provided as a Source Data file.

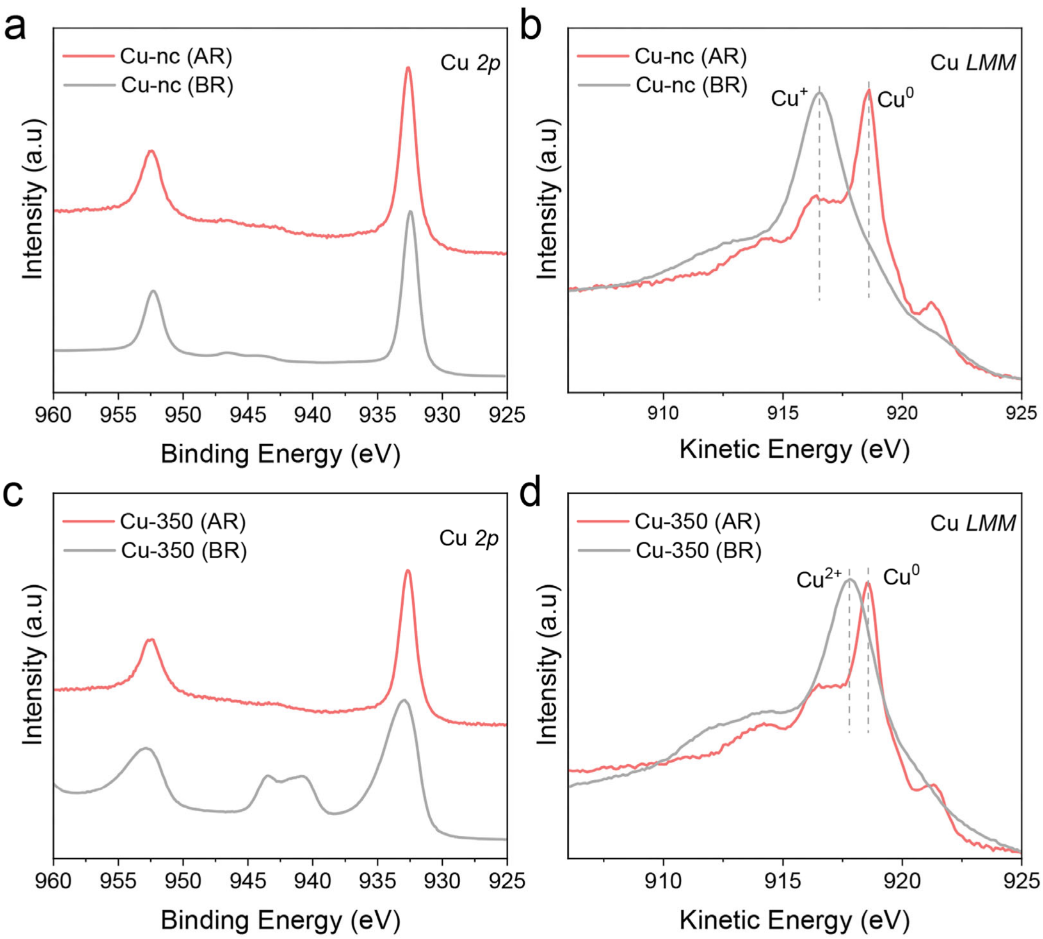

Supplementary Figure 20 | Quasi *in situ* Cu (a,c) 2p and (b,d) LMM XPS spectra of Cu-nc (a,b) and Cu-350 (c,d) catalysts before (grey) and (red) after CO electrolysis for 1 h at 2.6 V. Source data are provided as a Source Data file.

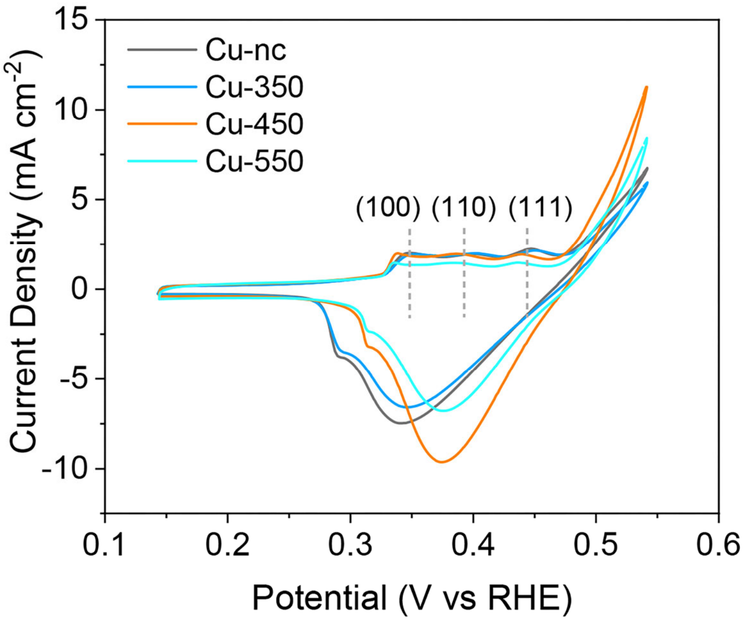

Supplementary Figure 21 | OH^- adsorption spectra measurements in 1.0 M KOH over Cu-nc and Cu-*x* catalysts at a scan rate of 20 mV s^{-1}. Source data are provided as a Source Data file.

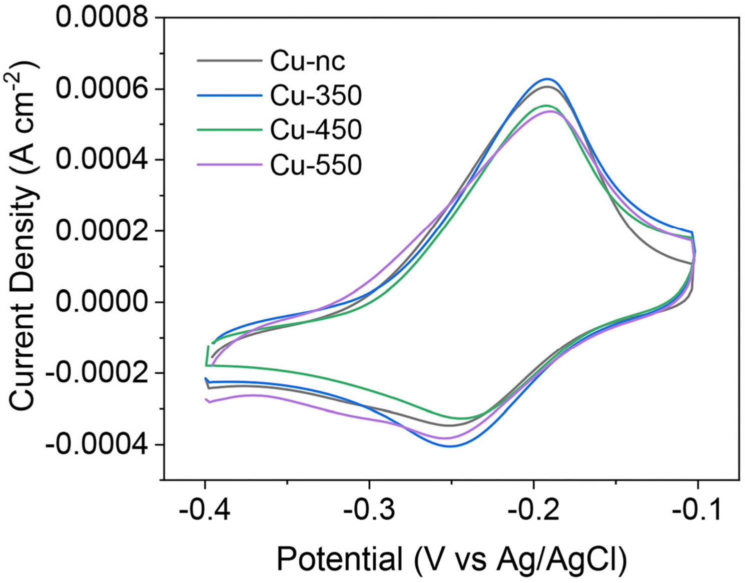

Supplementary Figure 22 | CVs for Cu-nc and Cu-*x* catalysts measured in 0.1 M $HClO_4$ + 0.001 M $Pb(ClO_4)_2$ in a H-cell at a scan rate of 10 mV s^{-1}. Source data are provided as a Source Data file.

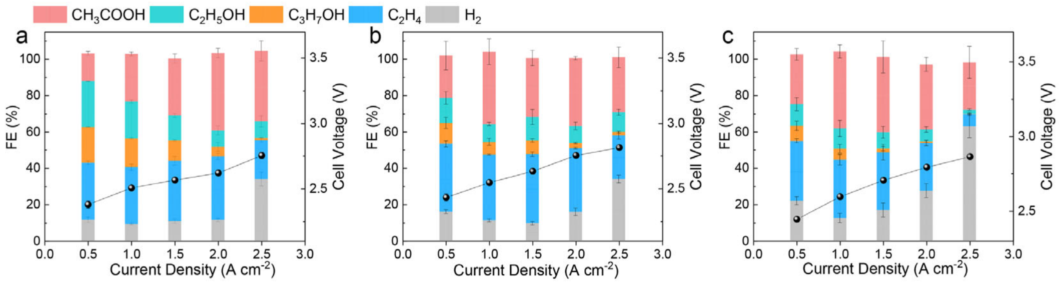

Supplementary Figure 23 | Faradaic efficiencies and cell voltages as a function of current density over Cu-350 (a), Cu-450 (b), and Cu-550 (c) catalysts. The error bars represent standard error of the mean and are made based on three fully separate and identical measurements. Source data are provided as a Source Data file.

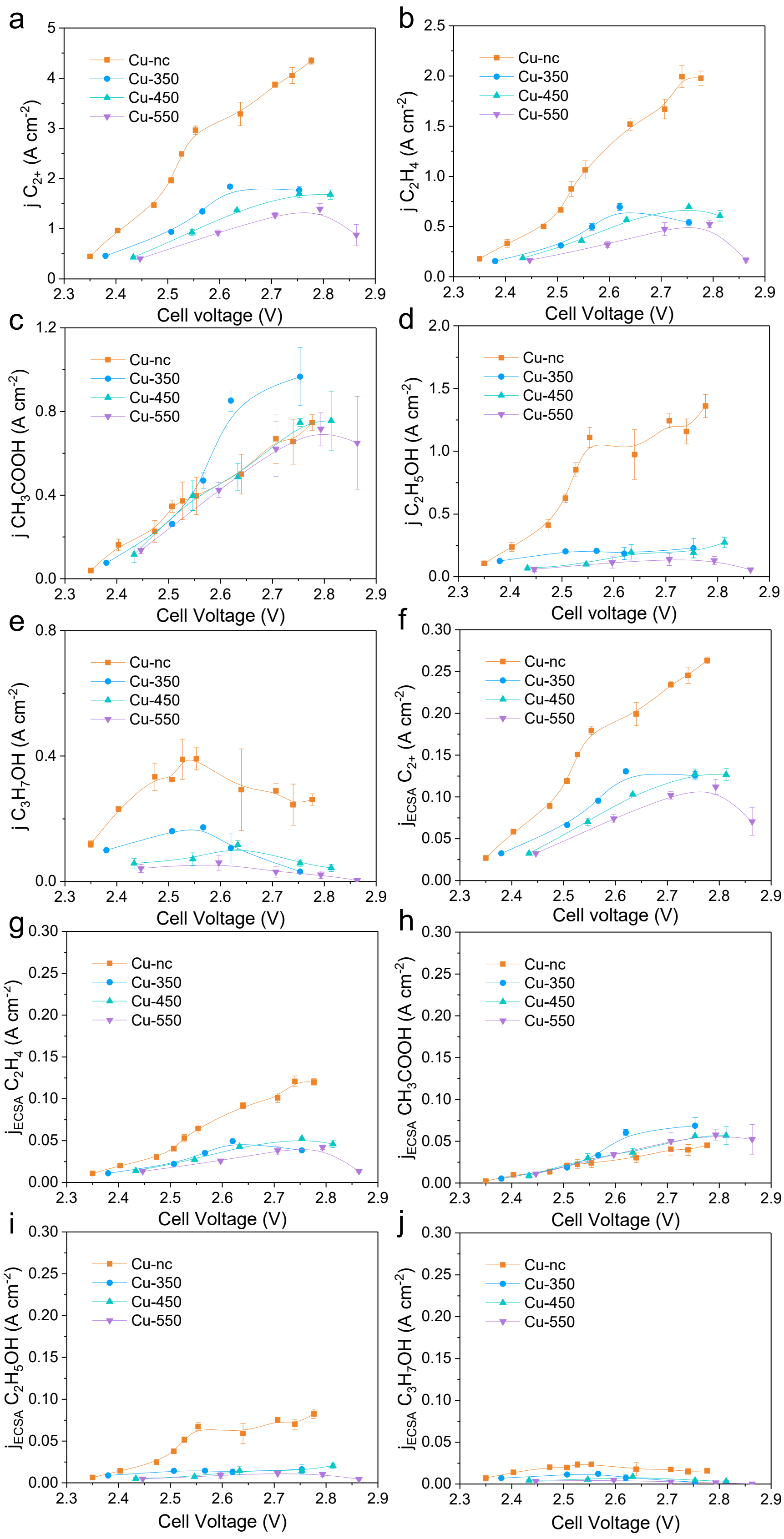

Supplementary Figure 24 | Geometric and ECSA-normalized partial current densities of C_{2+} (a, f), ethylene (b, g), acetate (c, h), ethanol (d, i), and n-propanol (e, j) as a function of cell

voltage over Cu-nc and Cu-*x* catalysts. The error bars represent standard error of the mean and are made based on three fully separate and identical measurements. Source data are provided as a Source Data file.

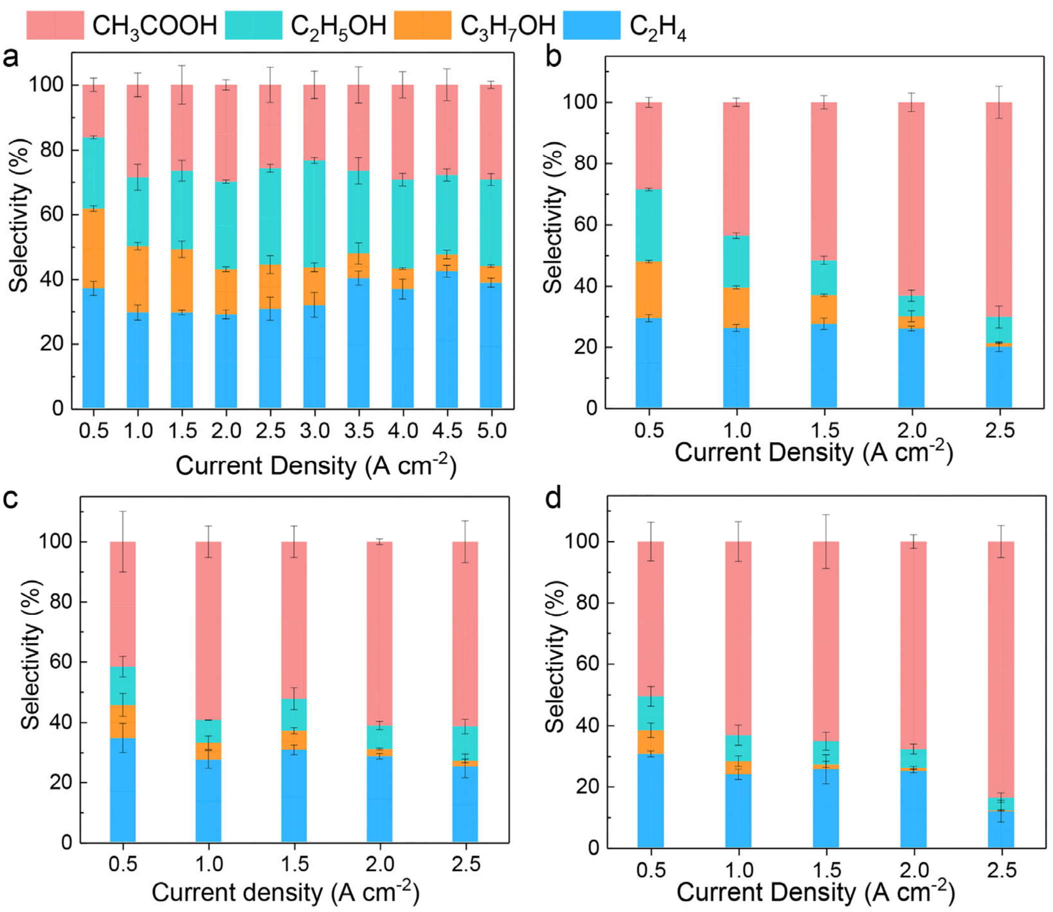

Supplementary Figure 25 | Carbon selectivity as a function of current density over Cu-nc (a), Cu-350 (b), Cu-450 (c), and Cu-550 (d) catalysts. The error bars represent standard error of the mean and are made based on three fully separate and identical measurements. Source data are provided as a Source Data file.

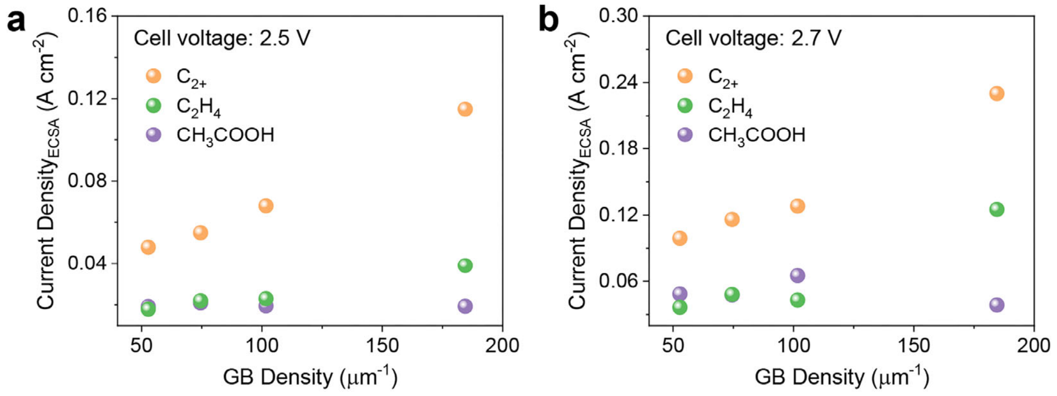

Supplementary Figure 26| Correlations between ECSA-normalized C_{2+}/ethylene/acetate partial current densities and GB density at a cell voltage of (a) 2.5 and (b) 2.7 V. Source data are provided as a Source Data file.

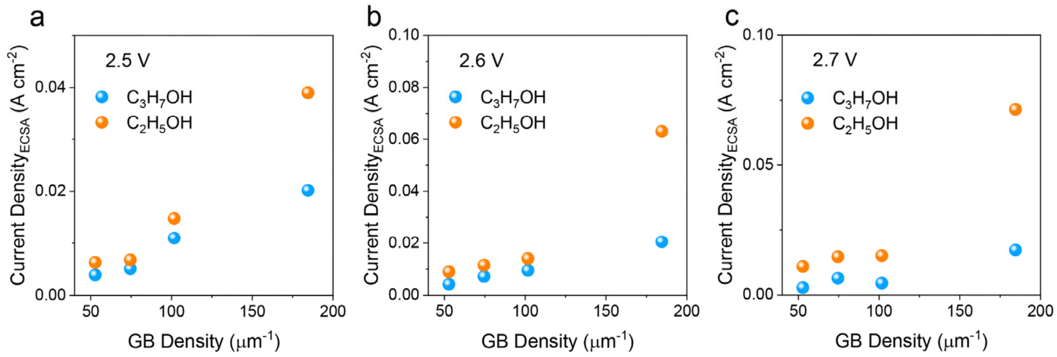

Supplementary Figure 27 | Correlations between ECSA-normalized ethanol and n-propanol partial current densities and GB density after electrolysis at a cell voltage of (a) 2.5, (b) 2.6, and (c) 2.7 V. Source data are provided as a Source Data file.

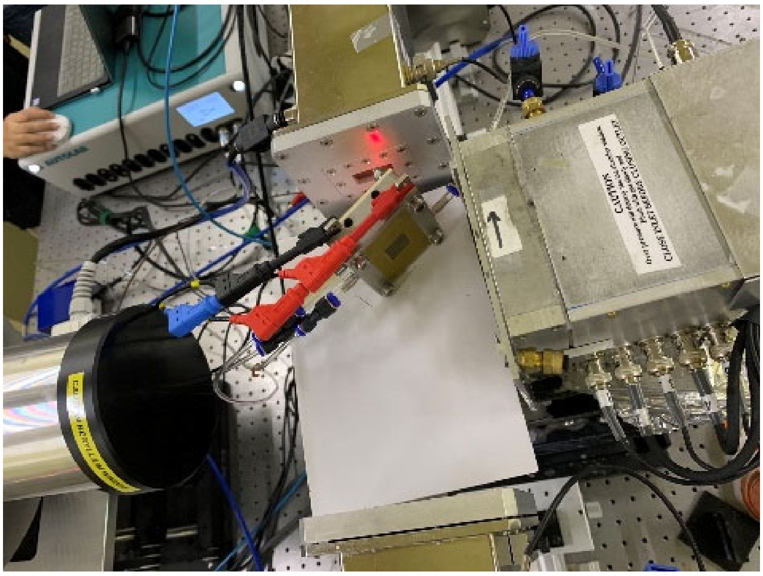

Supplementary Figure 28 | Photograph of operando XAS experiments conducted in a home-made MEA cell.

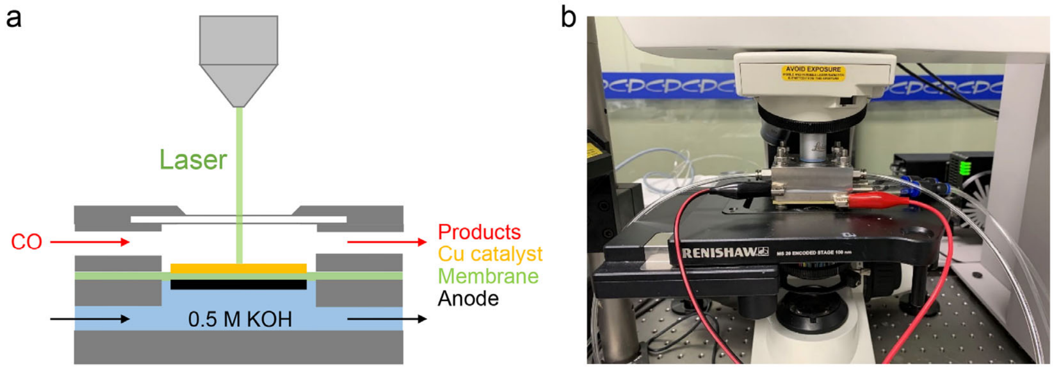

Supplementary Figure 29 | (a) Schematic of operando Raman spectroscopy measurements. (b) Photograph of operando Raman experiments conducted in a home-made MEA cell.

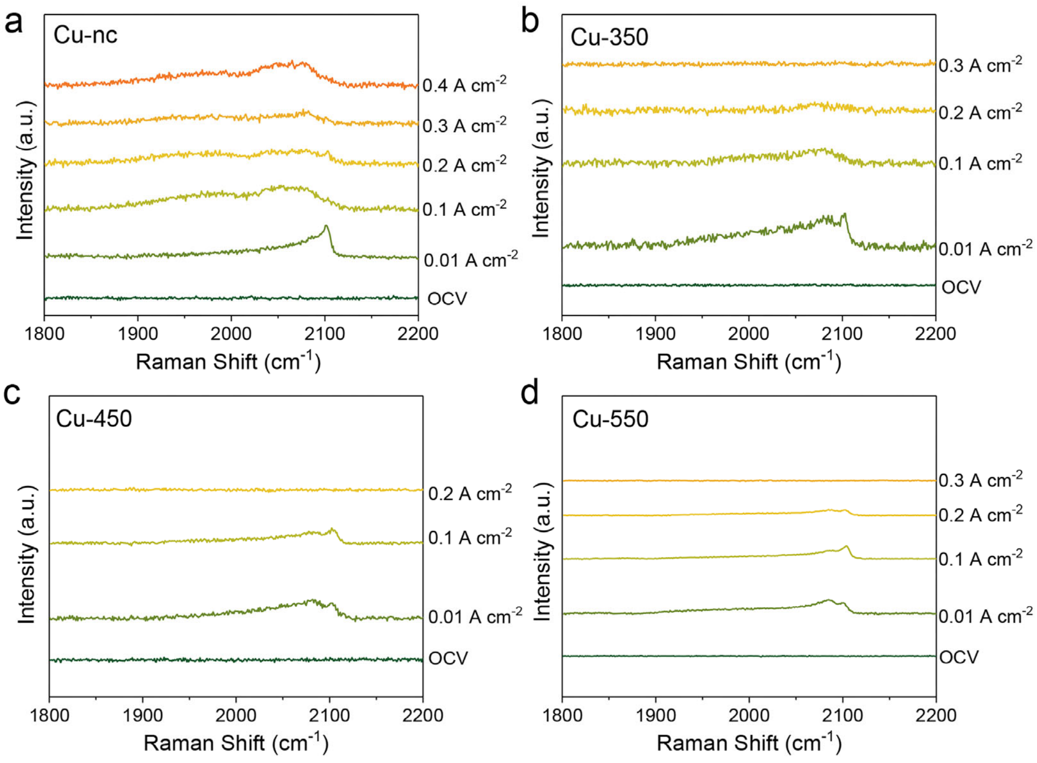

Supplementary Figure 30 | Operando Raman spectra over Cu-nc (a), Cu-350 (b), Cu-450 (c), and Cu-550 (d) catalysts under a range of applied current densities in a home-made MEA cell. Source data are provided as a Source Data file.

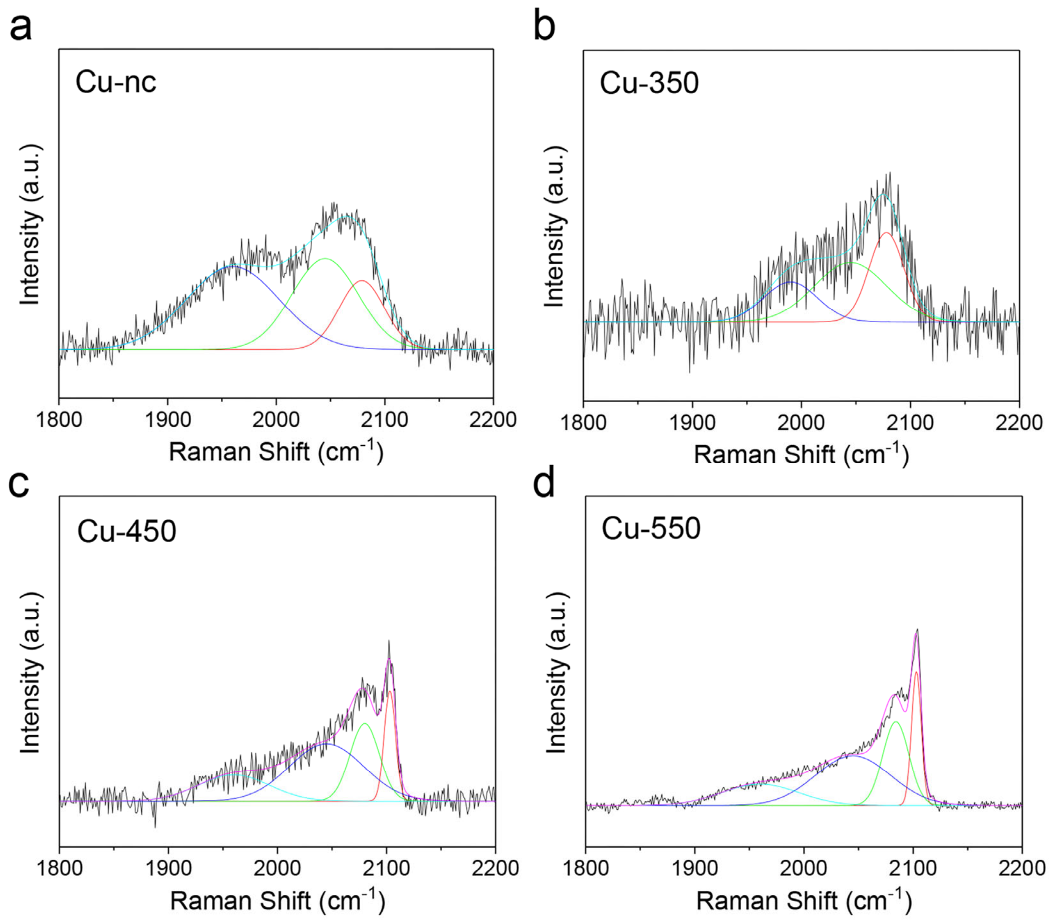

Supplementary Figure 31 | Gaussian fitting of operando Raman spectra for $*CO_{bridge}$ and $*CO_{atop}$ over Cu-nc (a), Cu-350 (b), Cu-450 (c), and Cu-550 (d) catalysts at 0.1 A cm^{-2}. Fitting results were summarized in Supplementary Table 7. Source data are provided as a Source Data file.

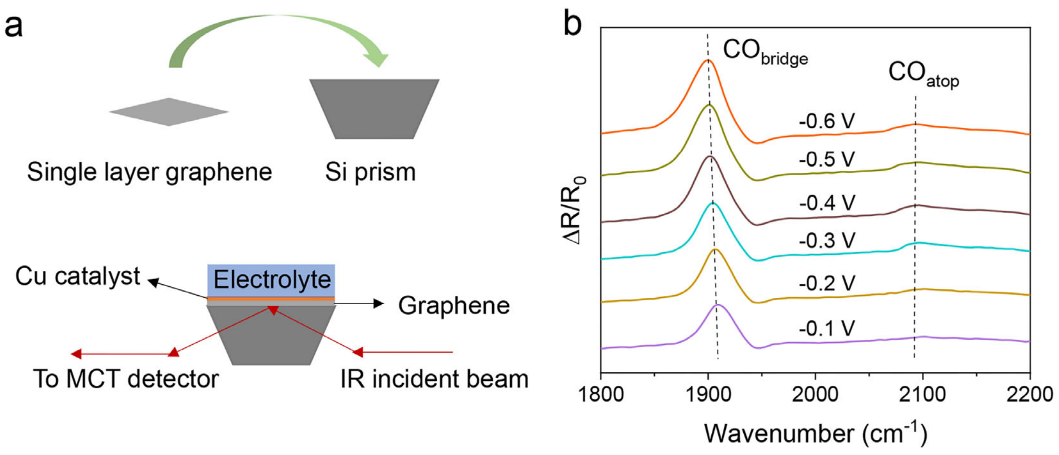

Supplementary Figure 32 | (a) Schematic of in situ ATR-FTIR spectroscopy measurements. (b) In situ ATR-FTIR spectra of Cu-nc catalyst under CO electrolysis at different potentails (versus RHE). The presence of $*CO_{bridge}$ indicates strong CO binding over Cu-nc catalyst. Source data are provided as a Source Data file.

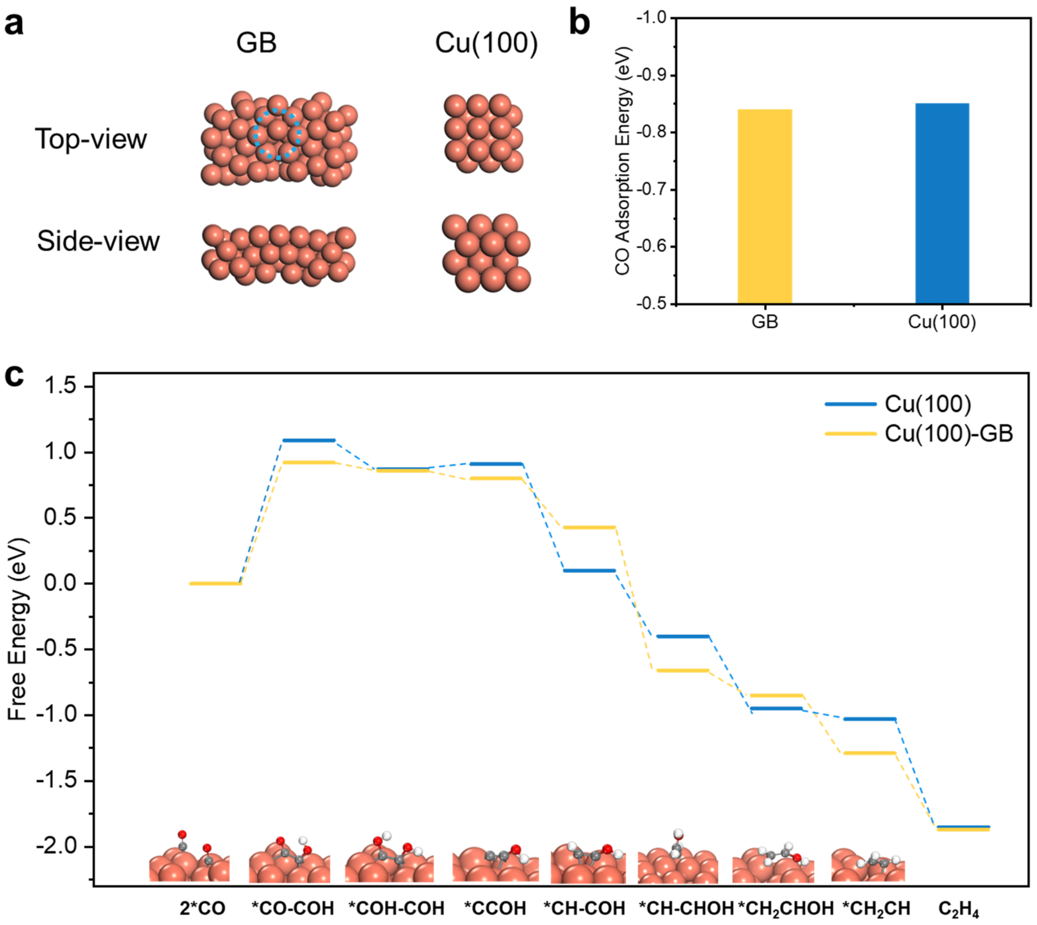

Supplementary Figure 33 | (a) Atomic configuration, (b) CO adsorption energy, and (c) free energy profile for ethylene production on GB and Cu(100). Source data are provided as a Source Data file.

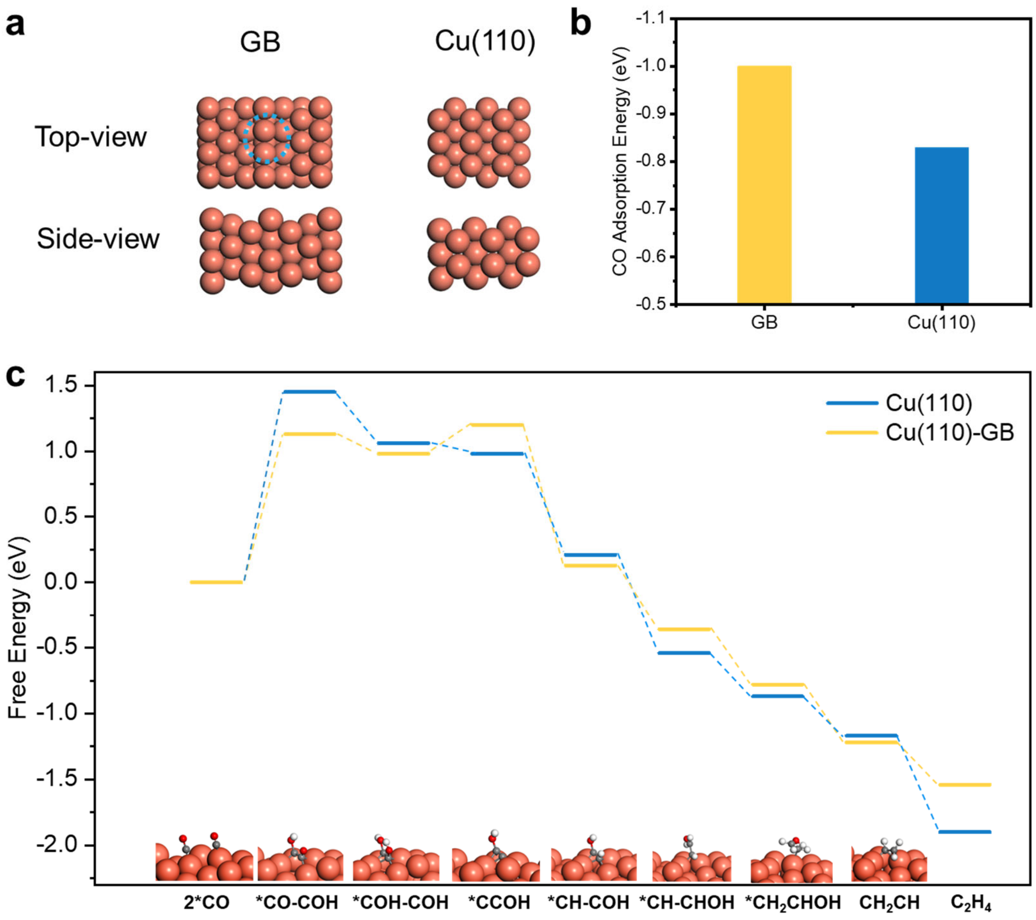

Supplementary Figure 34 | (a) Atomic configuration, (b) CO adsorption energy, and (c) free energy profile for ethylene production on GB and Cu(110). Source data are provided as a Source Data file.

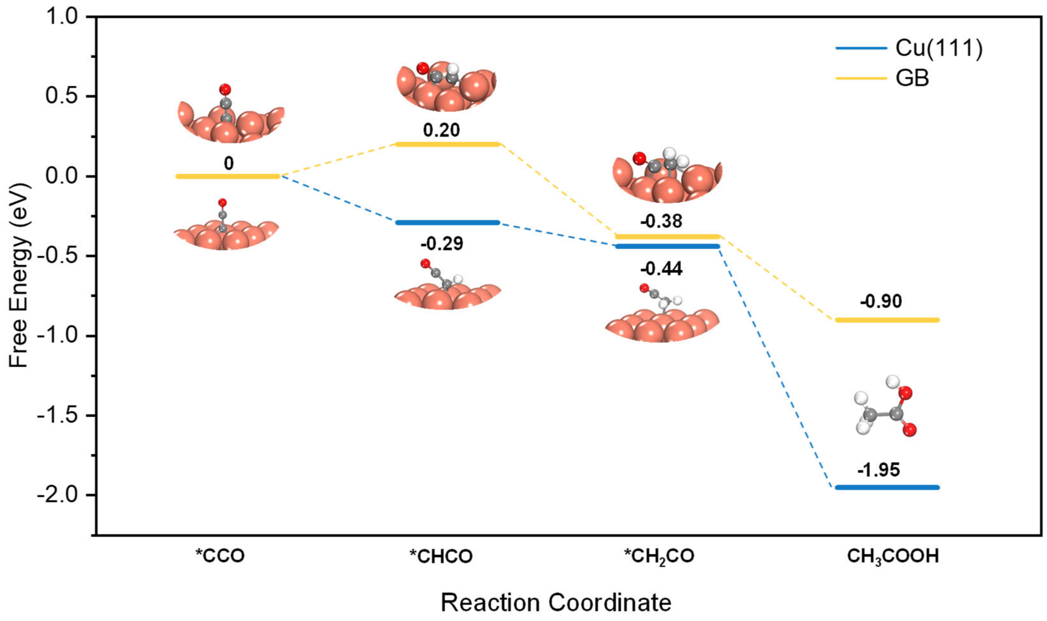

Supplementary Figure 35 | Free energy profile of CH_3COOH production on GB and Cu(111). Source data are provided as a Source Data file.

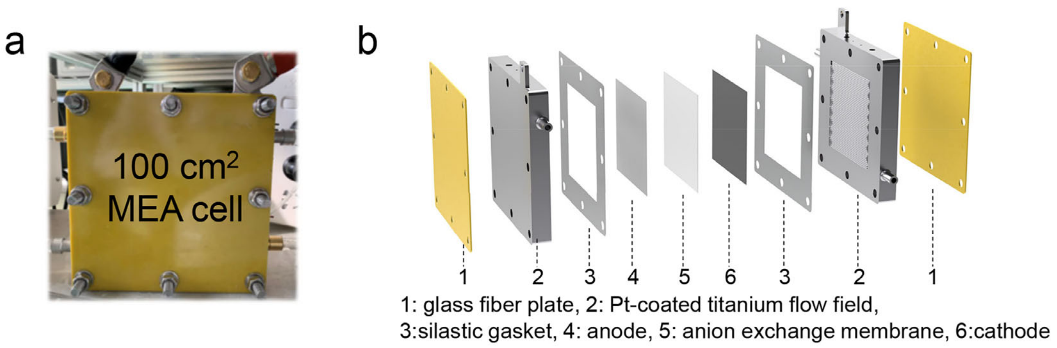

Supplementary Figure 36 | (a) Photograph of 100-cm^2 MEA electrolyzer. (b) Schematic of 100-cm^2 MEA electrolyzer.

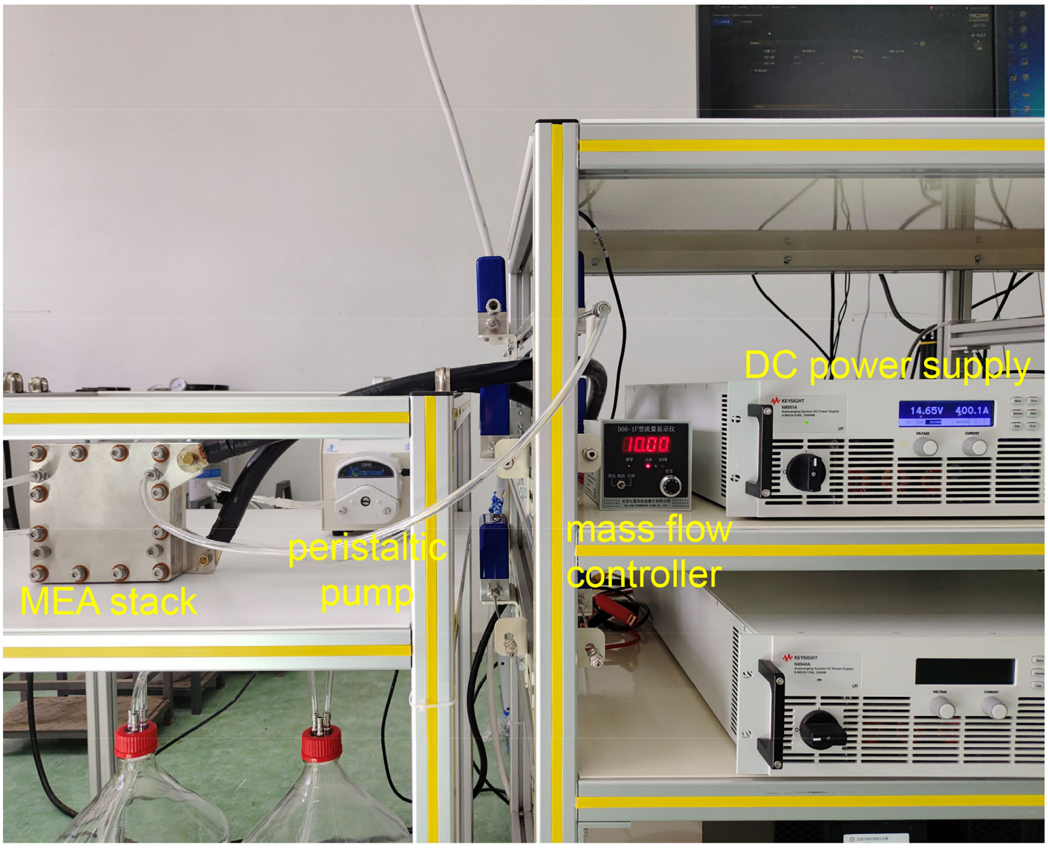

Supplementary Figure 37 | Photograph of the scale-up setup for electrolyzer stack measurement.

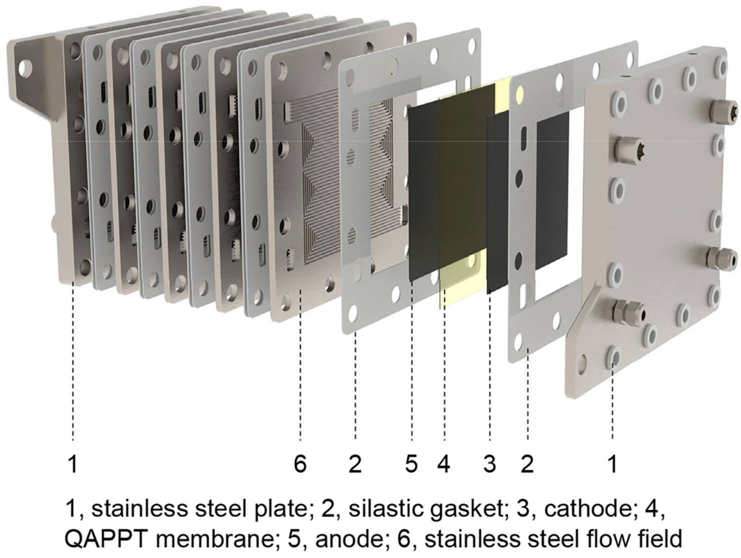

Supplementary Figure 38 | Schematic of electrolyzer stack with five 100-cm^2 MEAs in this work.

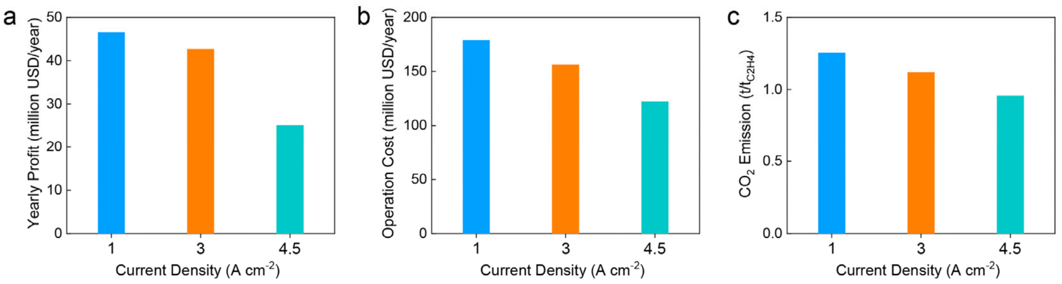

Supplementary Figure 39 | (a) Yearly profit, (b) operation cost, and (c) CO_2 emission for the production of 100 t/day ethylene calculated based on reported CO electrolysis performance in this work. Source data are provided as a Source Data file.

Supplementary Table 2 | Performances of CO electrolysis to C_{2+} products over recently reported catalysts.

Catalyst	Electrolyzer	Electrolyte	j_{C2+} (A cm^{-2})	C_{2+}FE (%)	Ref.
Cu-Pd	flow cell	1.0 M KOH	0.576	95	18
Cu-HDD	flow cell	1.0 M KOH	0.727	90	9
$Ag_2Cu_2O_3$	flow cell	1.0 M $CsHCO_3$	0.55	91.7	19
Pd-doped Cu	flow cell	1.0 M KOH	0.637	91.7	20
Cu nanosheets	flow cell	2.0 M KOH	0.182	60.6	21
Cu	flow cell	1.0 M KOH	1.41	93	8
OD-Cu	flow cell	1.0 M KOH	0.63	91	22
Cu:Py:SSC	MEA cell	3.0 M KOH	0.174	82	23
Cu-s	MEA cell	2.0 M KOH	1.34	84	24
CuO nanosheet	MEA cell	1.0 M KOH	3.12	90	15
**Cu-nc**	**MEA cell**	**0.5 M KOH**	**3.87**	**96.7**	**This work**
**Cu-nc**	**MEA cell**	**0.5 M KOH**	**4.05**	**90**	**This work**
**Cu-nc**	**MEA cell**	**0.5 M KOH**	**4.35**	**87**	**This work**

Supplementary Table 3 | Thermocatalytic performances of CO hydrogenation to C_{2+} products over recently reported catalysts and electrocatalytic performances of CO electrolysis to C_{2+} products over Cu-nc catalyst in this work.

Catalyst	Reaction conditions			X_{CO} (%)	Conv. rate (mL min^{-1})	S_{C2+} (%)	Y_{C2+} (%)	S_{CO2} (%)	S_{CH4} (%)	Ref.
	T (°C)	P (MPa)	F_{CO} (mL min^{-1})							
CoMnC/PDVB	250	0.1	19.2	63.5	12.2	51.1	32.4	46.3	2.6	5
$ZnCrO_x$/SAPO-34	400	2.5	10	17	1.7	57.8	9.8	41	1.1	3
Fe-Zn-0.81Na	340	2	4.8	77.2	3.7	66.5	51.3	23.8	9.7	25
FeMn@Si	320	3	11	56.1	6.1	77.5	43.4	13	9.5	4
$Co_{0.5}Mn$/AC	220	3	22.2	40.5	9.1	89.6	36.2	1.8	8.6	26
CoMn	250	0.1	16.7	31.8	5.3	50	15.9	47.3	2.6	27
Fe/α-Al_2O_3	340	2	22.5	80	18	53.4	42.7	40	6.6	28
$ZnAl_2O_4$/SAPO-34	400	3	10	24	2.4	54	13	44	1.9	29
Co@SiO_2-873	210	2	20	15.8	3.1	94.7	15	N.G.[a]	5.3	30
$ZnAlO_x$/SAPO-34	390	4	N.G.	6.9	N.G.	63.5	4.3	33.1	3.3	31
$ZnCrO_x$/AIPO-18	390	10	N.G.	49.3	N.G.	50.2	24.7	48.6	0.7	32
**Cu-nc**	**25**	**0.1**	**76**	**85.6**	**65.1**	**100**	**85.6**	**0**	**0**	**This work**

N.G. stands for Not Given.

F_{CO} denotes the inlet CO flow rate which is calculated by $GHSV \times m_{catalyst} \times V_{CO}\%$ for thermocatalytic process.

X_{CO} denotes single-pass conversion of inlet CO.

S_{C2+}, S_{CO2}, S_{CH4}, denotes the selectivity of C_{2+}, CO_2, CH_4 products with a sum of 100%.

Conv. rate denotes the flow rate of converted CO which is calculated by $F_{CO} \times X_{CO}$.

Y_{C2+} denotes the yield of C_{2+} products which is calculated by $\frac{X_{CO} \times S_{C2+}}{100}$

Supplementary Table 4 | GB lengths, surface area of Cu nanoparticles and GB densities (length per unit surface area) of Cu-nc and Cu-x catalysts before CO electrolysis.

Sample	Cu-nc (BR)			Cu-350 (BR)			Cu-450 (BR)			Cu-550 (BR)		
	$\sum L$	$\sum S_i$	$\frac{\sum L}{\sum S_i}$	$\sum L$	$\sum S_i$	$\frac{\sum L}{\sum S_i}$	$\sum L$	$\sum S_i$	$\frac{\sum L}{\sum S_i}$	$\sum L$	$\sum S_i$	$\frac{\sum L}{\sum S_i}$
a	118.4	474.575	249.5	43.246	462.3	93.5	74.35	1017.2	73.1	47.85	1144.67	41.8
b	69.1	356.183	194	55.63	443.2	125.5	70.01	1067.75	65.6	86.97	1195.7	72.7
c	66.4	383.6	173.1	25.688	367.78	69.8	37.06	610.6	60.7	32.72	624.34	52.4
d	84.85	429.3	197.6	46.2	597.7	77	48.71	1081	45.1	21.5	558.1	38.5
e	60	269	223	47.3	476.3	99.3	47.14	838	56.2	28.97	876.8	33
f	73	301	242.5	61.3	539.96	113.5	31.9	631.7	50.5	42.1	646.2	65.2
g	61.8	311.6	198.3	61.58	517.3	119	62.6	961.1	65.1	40.9	997.8	41
h	93	536	173.5	76.06	666.8	114	65.6	1062	61.7	25.6	1038.1	24.67
i	30.5	147	207.5	45.4	398.5	113.9	72.8	1016	71.7	52.21	714.9	73
j	43.36	236.4	183.4	82	919	89.2	69.2	763.6	90.6	72.51	1006.8	72

Supplementary Table 5 | GB lengths, surface area of Cu nanoparticles and GB densities (length per unit surface area) of Cu-nc and Cu-*x* catalysts after CO electrolysis.

Sample	Cu-nc (AR)			Cu-350 (AR)			Cu-450 (AR)			Cu-550 (AR)		
	$\sum L$	$\sum S_i$	$\frac{\sum L}{\sum S_i}$	$\sum L$	$\sum S_i$	$\frac{\sum L}{\sum S_i}$	$\sum L$	$\sum S_i$	$\frac{\sum L}{\sum S_i}$	$\sum L$	$\sum S_i$	$\frac{\sum L}{\sum S_i}$
a	162.17	853.1	190.1	56.9	590	96	37.6	684	55	54.4	677.3	80
b	342.5	2151	159.2	112.7	996.5	113	62.1	573.78	108	12.66	619.3	20.4
c	308.6	1820.5	169.58	149.9	1464	102	66.2	715.05	92	56.4	710	79.4
d	412.7	2577	160.1	87.5	671.8	130.2	42.1	651.93	64	24.55	519.68	47.2
e	361	1904	189.6	61.48	705.4	87.16	22.36	469.83	47.6	40	896.3	44.6
f	378.1	1916	197.3	80.6	689.2	116.9	26.72	639.27	41	70	1250	56.2
g	353.9	1970	179.6	111	984.27	112.7	40.6	615.63	65	30	535	56
h	412.6	1790	230.5	61.89	1171	52.9	41.22	484.8	85	30.38	711.34	42.7
i	1232.4	7030	175.3	73.24	982.17	74.57	70.45	722.79	97	58.8	1091	53.9
j	377.6	1949	193.7	90.7	686.8	132.1	49.49	541.3	91	56.73	1184.9	47.88

Supplementary Table 6│ GB densities of Cu-nc and Cu-x catalysts before and after CO electrolysis. The error bars represent standard error of the mean and are made based on fully separate and identical measurements.

**Sample**	**GB density (μm^{-1})**
Cu-nc (BR)	204.2±25.3
Cu-350 (BR)	101.4±17.7
Cu-450 (BR)	64.0±12.1
Cu-550 (BR)	51.4±17.2
Cu-nc (AR)	184.5±19.9
Cu-350 (AR)	101.7±23.6
Cu-450 (AR)	74.5±21.7
Cu-550 (AR)	52.8±16.5

Supplementary Table 7│ Roughness factors determined by Pb UPD measurements. The Pb stripping charge was obtained by integrating stripping peak located at −0.30 ~ −0.10 V vs. Ag/AgCl. The corresponding roughness factor was obtained by dividing the measured Pb stripping charge of Cu-nc and Cu-*x* with that of an electropolished Cu foil. The Pb stripping charge of an electropolished Cu foil was 310 μC cm^{-2}, according to the literature[46].

Electrode	Lead stripping charge (μC cm^{-2})	Roughness factor
Cu-nc	5121	16.51
Cu-350	4370	14.09
Cu-450	4112	13.26
Cu-550	3854	12.43
Cu foil	310[1]	1

Supplementary Table 8 | Fitting results of $*CO_{bridge}$ and $*CO_{atop}$ for operando Raman spectra over Cu-nc and Cu-*x* catalysts at 0.1 A cm^{-2}.

**Catalyst**	**Cu-nc**	**Cu-350**	**Cu-450**	**Cu-550**
$*CO_{bridge}$: 1900–2000 cm^{-1}	45.7%	22.4%	19.3%	18.5%
$*CO_{atop}$: ~2045 cm^{-1}	35.8%	44.9%	44.2%	42.2%
$*CO_{atop}$: ~2078 cm^{-1}	18.4%	32.5%	23.5%	24.7%
$*CO_{atop}$: ~2102 cm^{-1}	0	0	12.8%	14.5%
Total $*CO_{atop}$ [a]	54.3%	77.5%	80.6%	81.4%
HFB-$*CO_{atop}$ [b]	18.4%	32.5%	36.4%	39.2%
$*CO_{bridge}$/$*CO_{atop}$ [c]	0.84	0.29	0.24	0.23
LFB/HFB-$*CO_{atop}$ [d]	1.93	1.38	1.21	1.07

[a] Total $*CO_{atop}$: $*CO_{atop}$ at 2045, 2078 and 2102 cm^{-1}.

[b] HFB-$*CO_{atop}$: High-frequency-band-$*CO_{atop}$ at 2078 and 2102 cm^{-1}.

[c] $*CO_{bridge}$/$*CO_{atop}$:

$$*\,\mathrm{CO}_{bridge}/*\,\mathrm{CO}_{atop} = \frac{*\,\mathrm{CO}_{bridge}\ \text{at}\ 1900-2000\ \mathrm{cm}^{-1}}{*\,\mathrm{CO}_{atop}\ \text{at}\ 2045, 2078\ \text{and}\ 2102\ \mathrm{cm}^{-1}}$$

[d] LFB/HFB-$*CO_{atop}$:

$$\mathrm{LF/HF}*\,\mathrm{CO}_{atop} = \frac{*\,\mathrm{CO}_{atop}\ \text{at}\ 2045\ \mathrm{cm}^{-1}}{*\,\mathrm{CO}_{atop}\ \text{at}\ 2078\ \text{and}\ 2102\ \mathrm{cm}^{-1}}$$

Supplementary Table 9 | The calculated free energy corrections for adsorbates. All values are given in eV.

**Adsorbate**	**Zero Point Energy**	$\int C_p dT$	**TS**
2*CO	0.34	0.17	0.34
*COCOH	0.67	0.15	0.30
*COH-COH	1.02	0.14	0.30
*CCO	0.34	0.09	0.19
*CCOH	0.59	0.11	0.20
*CHCOH	0.89	0.10	0.19
*CHCO	0.60	0.11	0.24
*CHCHOH	1.22	0.12	0.27
*CH_2CO	0.87	0.14	0.31
*CH_2CHOH	1.52	0.14	0.30
*CH_2CH	1.07	0.09	0.17
